# The current status of geriatric oncology in India

**DOI:** 10.3332/ecancer.2023.1595

**Published:** 2023-08-31

**Authors:** Vanita Noronha, Abhijith Rajaram Rao, Anant Ramaswamy, Anita Kumar, Anupa Pillai, Ratan Dhekale, Jyoti Krishnamurthy, Akhil Kapoor, Shreya Gattani, Arshiya Sehgal, Sharath Kumar, Renita Castelino, Sarika Mahajan, Anuradha Daptardar, Lekhika Sonkusare, Jayita Deodhar, Nabila Ansari, Manjusha Vagal, Purabi Mahajan, Shivshankar Timmanpyati, Manjunath Nookala, Ankita Chitre, Prem Naganath Narasimhan, Joyita Banerjee, Vikram Gota, Shripad Banavali, Rajendra A Badwe, Kumar Prabhash

**Affiliations:** 1 Department of Medical Oncology, Tata Memorial Centre, Homi Bhabha National Institute, Mumbai 400012, India; 2 Department of Geriatric Medicine, All India Institute of Medical Science, New Delhi 110023, India; 3 Department of Medical Oncology, Mahamana Pandit Madan Mohan Malviya Cancer Center & Homi Bhabha Cancer Hospital, Varanasi 221005, India; 4 Department of Clinical Pharmacology, Advanced Centre for Treatment Research and Education in Cancer, Navi Mumbai 410210, India; 5 Department of Physiotherapy, Tata Memorial Centre, Homi Bhabha National Institute, Mumbai 400012, India; 6 Department of Psycho-oncology, Tata Memorial Centre, Homi Bhabha National Institute, Mumbai 400012, India; 7 Department of Occupational Therapy, Tata Memorial Centre, Homi Bhabha National Institute, Mumbai 400012, India; 8 Department of Digestive Diseases and Clinical Nutrition, Tata Memorial Centre, Homi Bhabha National Institute, Mumbai 400012, India; 9 Department of Physiotherapy, Mahamana Pandit Madan Mohan Malviya Cancer Center & Homi Bhabha Cancer Hospital, Varanasi 400012, India; 10 Consultant Geriatrician, Jaslok Hospital and Research Center, Mumbai 400026, India; 11 Venu Geriatric Care Centre, Venu Charitable Society, New Delhi 110017, India; 12 Department of Surgical Oncology, Tata Memorial Centre, Homi Bhabha National Institute, Mumbai 400012, India

**Keywords:** India, LMIC, collaboration, research, education, training, older, elderly

## Abstract

Geriatric oncology in India is relatively new. The number of older persons with cancer is increasing exponentially; at our institution, 34% of patients registered are 60 years and over. Apart from the Tata Memorial Hospital in Mumbai, there are currently no other Indian centers that have a dedicated geriatric oncology unit. Geriatric assessments (GAs) are done sporadically, and older patients with cancer are usually assessed and treated based on clinical judgement. Challenges to increasing the uptake of GA include a lack of training/time/interest or knowledge of the importance of the GA. Other challenges include a lack of trained personnel with expertise in geriatric oncology, and a paucity of research studies that seek to advance the outcomes in older Indian patients with cancer. We anticipate that over the next 10 years, along with the inevitable increase in the number of older persons with cancer in India, there will be a commensurate increase in the number of skilled personnel to care for them. Key goals for the future include increased research output, increased number of dedicated geriatric oncology units across the country, India-specific geriatric oncology guidelines, geriatric oncology training programs, and a focus on collaborative work across India and with global partners. In this narrative review, we provide a broad overview of the status of geriatric oncology in India, along with a description of the work done at our center. We hope to spark interest and provide inspiration to readers to consider developing geriatric oncology services in other settings.

## Introduction

Geriatric oncology in India is in its infancy. The majority of older Indian patients with cancer are not evaluated systematically with a validated geriatric assessment (GA), but rather are assessed using clinical judgement. Due to a severe shortage of persons skilled in geriatric oncology, and the lack of dedicated geriatric oncology units both in major academic centers as well as in corporate hospitals and private practices in India, the care provided to older Indian patients with cancer is likely to be sub-optimal [1]. Besides, several GA tools and scales that were developed in the West are not suitable for some older Indian patients, due to differences in cultural experiences, illiteracy, and varying life expectancies [2].

The functional status is a measure of the ability of an individual to perform tasks that maintain independence [3]. This functional status declines progressively with age, making older individuals vulnerable to stressors and rendering some incapable of living independently, functionally and socially [4]. There is heterogeneity in the ageing process, which further contributes to the complexity of treatment decisions. These factors result in age-related variations in treatment patterns and outcomes, potentially culminating in under- or overtreatment, which can influence both the risks of treatment toxicity, and survival [5]. Though the Eastern Cooperative Oncology Group (ECOG) performance status (PS), which is the traditional method used by oncologists to evaluate functional status globally, has been found to correlate moderately well with vulnerabilities in various geriatric domains, it cannot replace a formal GA [6]. Despite the broad scope of geriatric oncology, only a few Indian centers offer a formal geriatric oncology service, or the ability to provide specialised care to an older person with cancer.

There are myriad other issues plaguing geriatric oncology in India, including a lack of teaching programs, almost no research studies that either include older persons with cancer or focus on issues important to older patients with cancer, and a lack of an inter-institutional and pan-India collaborative effort to advance the care of older Indian patients with cancer. Many of these issues are not unique to India, but are global phenomena. We explore the current status of geriatric oncology in India, the challenges, strengths and opportunities and suggest the key focus areas over the course of the next decade. Through this review article, we hope to inspire at least some readers to build up geriatric oncology services at their clinics, and establish centers of excellence in geriatric oncology at various institutions across India.

## Epidemiology of older adults in India

In 2010, 524 million individuals globally were aged 65 years or older. By the year 2050, the World Health Organisation has predicted that 1.5 billion people will be 65 years or older [7]. Nearly 80% of these older individuals will be living in low-and middle-income countries [8]. This will have a tremendous impact on the global cancer burden. India is also witnessing a demographic transition. In 2011, older persons constituted 8.6% of India’s total population, equalling 103 million [9]. This proportion is expected to increase exponentially and is projected to reach 19.5% in 2050, equalling 319 million people. The proportion of the oldest-old, i.e., those aged 75 years and over, is projected to rise by 340% [10, 11].

Advancing age is a risk factor for the development of malignancy, with persons over 65 years accounting for 60% of newly diagnosed malignancies and 70% of cancer deaths. These mainly include cancers of the breast, lung, prostate, cervix, esophagus and ovary [12]. Globally, there are geographic and economic factors that affect the number of older people living with and surviving cancer, but data show a common trend that a large proportion of people with cancer are older [13, 14]. The Indian Council of Medical Research National Cancer Registry Program estimates that the number of cancer diagnoses will increase from 1.39 million in 2020 to 1.57 million by 2050 [15]. Thus, the number of older persons with cancer is expected to rise exponentially in India over the next few years. Data from our hospital-based registry at the Tata Memorial Hospital in Mumbai revealed that between January 2019 and December 2021, there were 29,768 patients with cancer registered who were over 60 years old among the total pool of 87,101 newly registered patients, i.e., 34.2% of the total number of patients with cancer registered at our institute were 60 years or older [unpublished data].

## Uptake of the GA in India

We conducted a survey among 100 physicians to assess the situation at the ground level regarding the extent of knowledge and practice of GA in India [16]. Although 99% of respondents cared for older patients with cancer, with 87% caring for 10 or more per week, 51% did not routinely perform a GA. Almost 70% of the survey participants relied on ‘intuition’ to formulate the management plan; less than 10% used validated tools. Additionally, 44% were unaware of the American Society of Clinical Oncology (ASCO) geriatric oncology guidelines. Barriers to performing the GA routinely included lack of time, staff, awareness, resources and space, and an uncertainty regarding which GA tools to use. The results of our survey underlined the need to devise a tailored GA tool in India for screening, evaluation, and management in limited resource settings [16].

## Oncologists’ perceptions of the need for assessing individual domains in the GA and worthwhile outcomes

We conducted a follow-up survey among 234 healthcare professionals (71% medical oncologists, 11.7% radiation oncologists, and 10.7% surgical oncologists) to understand the perceived relative importance of assessing various GA domains [17]. Over 99% participants thought that it was important to assess comorbidities, polypharmacy, medications, drug interactions and social support. Over 98% thought it important to assess function, falls, and quality of life (QoL). Approximately 97% thought that assessment of nutrition and cognition was important, while 93.6% thought that psychological evaluation was important. Regarding the goals of performing a GA, 90% hoped that it would lead to an improvement in QoL, 65.6% desired a decrease in toxicity, and 42.9% wished for survival prolongation. Regarding the relative importance of a decrease in treatment toxicity or improvement in QoL versus survival prolongation in older patients with cancer, 62.6% found toxicity + QoL more important, 36.9% found them as important and only 0.5% respondents felt that decreasing toxicity and improving QoL were less important than prolonging survival [17].

## Defining the age-cutoff for ‘old’

The answer to the question, ‘How old is old?’ varies based on multiple factors. For an individual, the factors are myriad; however, at the population level, the answer is straightforward and based on life expectancy and retirement age [18]. The International Society of Geriatric Oncology (SIOG) defines this age as 70 years, while the ASCO recommendations are for persons aged 65 years and older. When we started the geriatric oncology clinic at the Tata Memorial Hospital (TMH), in Mumbai, India, we evaluated patients aged >65 years based on the ASCO guidelines [19]. We later realised that the age cut-off to define ‘old age’ varies across cultures, and that using the age cutoff established by an American organisation was not appropriate for Indian patients. The average life expectancy in India is 70.4 years overall; 71.8 years for women, and 69.2 years for men [20], which is much lower than that of people living in Japan (85 years) or the United States (79 years). In Japan, the age cut-off for the geriatric population is considered to be 75 years, while in India, according to the National Program for Health Care of the Elderly (NPHCE) [21] established by the Government of India, an older person is defined as anyone who is 60 years or older. Hence the age cut-off in our geriatric oncology clinic was changed to 60 years [22]. This age cut-off is followed by other Indian clinicians as well [23, 24].

## Geriatric oncology service at the Tata Memorial Hospital

**Figure 1.**
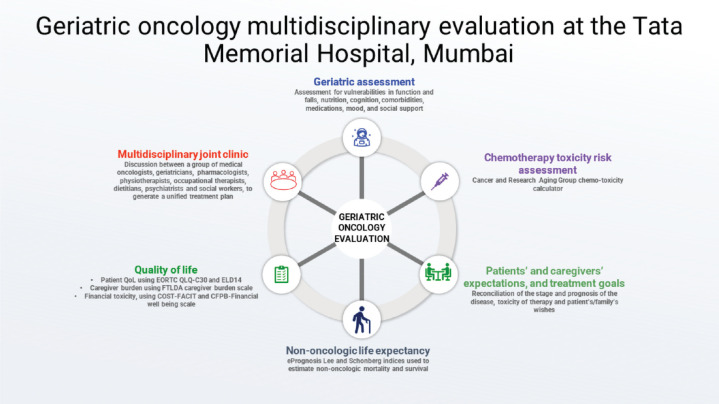
The process of evaluation and assessment in the geriatric oncology at the Tata Memorial Hospital in Mumbai, India.

**Figure 2.**
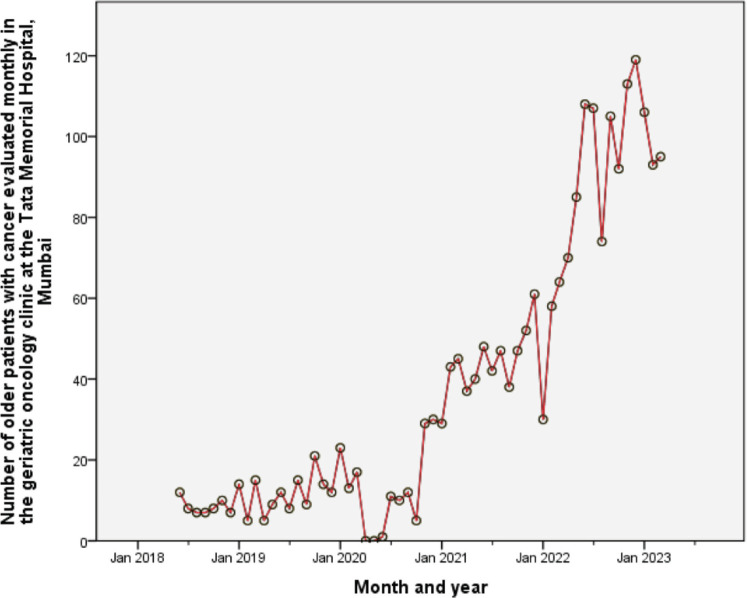
The progressive increase in the number of older patients with cancer evaluated monthly in the geriatric oncology clinic at the Tata Memorial Hospital (Mumbai, India) since inception on June 15, 2018.

TMH has a well-established geriatric oncology multidisciplinary service for patients aged 60 years and above with cancer. Starting on June 15, 2018, as a 1-day-a-week outpatient clinic, the service now functions daily, both for outpatients and inpatient consultations. Our goal is to perform detailed GAs and provide multidisciplinary, patient-specific care plans (Figure 1). In addition, we also run an academic teaching program and are attempting to bridge the research gap. The clinic is run by two medical oncologists; other members include a geriatric oncology fellow (trainee), senior residents, and patient coordinators. This multidisciplinary clinic also comprises clinical pharmacologists, geriatricians, onco-psychologists, physiotherapists, occupational therapists, dieticians, and social workers. Although initial referrals to our clinic were sparse, these have increased exponentially over time (except for the brief period when we closed the clinic from Apr to Jun 2020 during the peak of the COVID-19 pandemic), and currently, we assess approximately 20–30 patients a week. Between Jun 15, 2018, and May 12, 2023, we have assessed 2,322 patients (Figure 2).

## GA done at the Tata Memorial Hospital and details of the tools used

### Overall assessment

All patients referred to our geriatric oncology clinic undergo a detailed evaluation for the presence of vulnerabilities in various geriatric non-oncologic domains and caregiver burden. Patients are evaluated for geriatric syndromes (constipation, insomnia, lower urinary tract symptoms, urinary incontinence, osteoporosis, pressure sores), visual and hearing impairment, voice, and oral health. We assess chemotherapy toxicity risk using the Cancer and Aging Research Group (CARG) tool and estimate the non-oncologic life expectancy using ePrognosis. We also evaluate financial toxicity, and overall QoL (Table 1).

### Non-oncologic domains

The various GA tools used at our clinic, along with the cut-offs have been provided in Table 1.

### Blood tests

The patient's baseline haemoglobin, creatinine, albumin and sodium are noted, and the estimated glomerular filtration rate (eGFR) is calculated for all patients. Additionally, we determine the patient's neutrophil-lymphocyte ratio (NLR) considering it a prognostic inflammatory marker in older patients with cancer.

### Patients’ and caregivers’ goals of therapy

The patient's perspective regarding the intent of therapy (curative/palliative) and the patient's expectations from treatment and the patients’/primary caregivers' wishes regarding disclosure about the diagnosis/prognosis are discussed, and integrated into the care plan.

## Appropriateness of GA tools for older Indian adults

The GA has been developed in a predominantly western urban cohort of patients, and multiple aspects are culturally inappropriate for our Indian patients [2]. The problems with individual assessment using tools, such as instrumental activities of daily living (IADL) (roles and responsibilities within family vary diversely), mini-mental status examination (MMSE) (literacy, relocation to Mumbai for treatment, unawareness of dates according to the Gregorian calendar), mini-nutritional assessment (MNA) (lack of access to a weighing scale, thin body habitus), a cumulative illness rating scale for geriatrics (CIRS-G) (lack of strong primary healthcare systems, so no robust diagnosis and therapy of comorbidities), geriatric depression scale (GDS) and generalised anxiety disorder assessment-7 (GAD7) (culturally inappropriate questions) are prevalent. We have found that multiple portions of the GA are culturally inappropriate and could not be analysed or led to unreliable results [2]. The first step in tackling this issue was taken by Banerjee *et al* [54], who developed a short-35-item screening tool that would be appropriate for Indian patients. Further, they also identified suitable cut-off scores that correlated with survival, which could help streamline care in a resource-limited setting [55]. By reconciling the cultural differences and establishing a GA directed at Indian patients with cancer, we hope to be able to provide better service to our patients.

In addition to socio-cultural differences, phenotypic diversity cannot be ignored. The normal range of the body mass index (BMI) for the Asian population is lower as compared to that in western countries [56]. We identified that the cut-off TUG score of 12 seconds [19] was not as sensitive, and a lower cut-off score of 10 seconds was more appropriate for older Indian patients with cancer [57].

## Prevalence of non-oncologic vulnerabilities in older persons with cancer in India

In our geriatric oncology clinic at TMH, the median time to perform a GA is 50 minutes (IQR, 40–61) [58]. Despite the time-consuming process, we found that the GA was very valuable in detecting geriatric vulnerabilities. About 98% of our patients had vulnerabilities in at least one geriatric domain. The abnormalities noted were in the domains of comorbidities (79%), fatigue (77%), nutrition (65%), function and falls (52%), mood (32%), and cognition (18%) [58]. We also observed that 55% of the patients had polypharmacy, 80% were on potentially inappropriate medications (PIM), and nearly 23% were taking alternative medications (ayurvedic/naturopathic/homeopathic) [59]. Almost 60% needed referrals to a physiotherapist and occupational therapist, 69% required dietary intervention and 29% required evaluation and management by a psychologist/counsellor.

## Impact of the GA on the systemic therapy plan

Globally, it is well recognised that systematic evaluation of older patients with cancer with a GA is valuable and leads to better treatment choices, lowers toxicity and improves communication. However, conducting the GA is time-and labor-intensive, and resources are limited at most centers in India. At our institution, the GA led to a change in cancer-directed systemic therapy plan in 38.7% of cases, with the commonest change being treatment de-intensification in 32.1% of patients [60]. Thus, a significant proportion of our older patients with cancer are over-treated in cases where a GA is not performed.

## Geriatric oncology education programs

The Department of Medical Oncology at the Tata Memorial Hospital runs a 1-year geriatric oncology fellowship training program [61]. The program admits doctors trained in Internal Medicine, Geriatric Medicine, and Medical Oncology. The fellow undergoes rigorous training in patient assessment, care-plan development, academic learning, and research protocol development and implementation. The department also conducts Geriatric Oncology workshops at least twice a year to train oncologists and primary care physicians. The goal of such continuous medical education includes spreading awareness of geriatric oncology, imparting education regarding the principles of geriatric oncology, and training for performing GAs for older patients with cancer. The Tata Memorial Center has also partnered with SIOG to conduct an annual advanced course in geriatric oncology [62].

## Status of research in geriatric oncology in India

Due to the heterogeneous population and lack of data from older Indian patients with cancer, we generally need to extrapolate the results from studies done in other populations [63]. Broadly, we envision that the most impactful geriatric oncology research in India would include epidemiologic research to understand the demographic profile and various other epidemiologic parameters; tool validation research to test the appropriateness of assessment tools; implementation research to discover and test methods to increase the uptake/streamline or improve the efficiency of the GA; observational studies to understand the various aspects of geriatric oncology; therapeutic research aimed either at testing, cancer-directed therapy or treatment-related toxicities; and patient-centric research to understand and improve the experiences/perspectives/challenges faced by patients/caregivers (Table 2). A major challenge to conducting geriatric oncology research in India is the lack of collaboration. A limiting factor is the lack of centers that have geriatric oncology units and clinicians who have expertise in geriatric oncology. We have attempted to address this issue by forming a group of like-minded clinicians from various centers across India who have an interest in geriatric oncology. Several initiatives have been taken in this group to bridge the knowledge and research gap in geriatric oncology in India, some of which have been described in Table 2.

## Patient and caregiver support groups

Addressing the supportive care needs of an increasing number of older patients with cancer and their caregivers requires innovative strategies and delivery methods. Support groups bring together people who are going through or have gone through similar experiences. Online support groups are social spaces where people with shared interests can gain and share information and support. Spurred on by the COVID-19 pandemic and the increasing need for additional counselling/support/advice by patients and caregivers, in May 2022 we started an online support group for patients and caregivers of older patients with cancer. This is conducted with the help of a non-governmental organisation, and has been held on a virtual platform successfully every month. Along with patients and caregivers sharing their views and experiences, they are also educated regarding the importance of maintaining the patients' functionality, nutrition, cognitive capacity and mood.

## Action points for the next decade

In the coming decade, there will be an exponential increase in the number of older persons with cancer. In order to provide optimal support for these older and often frail patients, our focus will be on five high-impact areas, outlined in Table 3.

## Conclusion

There is a need for awareness of geriatric oncology among practicing physicians who care for older patients with cancer. Only a few institutes in India provide specialised geriatric oncology services. Recognising the importance and implementing such a service to optimally manage this vulnerable population is an urgent necessity. At the Tata Memorial Hospital in Mumbai, we have established a comprehensive multidisciplinary geriatric oncology service and are attempting to fill the gap in terms of providing specialised care to older patients with cancer, designing and implementing research studies that focus on this cohort of patients, and disseminating high-quality education to students and colleagues. Since the clinic was established in June 2018, we have accomplished a lot in terms of clinical service, education and research; however, much more needs to be done. More physicians, oncologists, geriatricians, and nursing staff need to be trained to perform GAs, interpret the results, and prepare tailored care plans. There is a need to develop and validate suitable and culturally appropriate tools to assess older Indian patients with cancer.

## Conflicts of interest

None.

## Funding

None.

## Figures and Tables

**Table 1.** Details of the tools used, assessments performed and questionnaires administered as a part of the GA at the Tata Memorial Hospital (Mumbai, India).

GA tool	Scale	Cut-off to define an abnormal value (vulnerability in the GA)
Function		
Activities of daily living (ADL)	Katz ADL [25]	<6
IADL	Lawton IADL [26]	Men <5 Women <8
Performance-based assay	Timed-up-and-go [27]	>10 seconds
Risk of falls	Single question: ‘How many falls have you had in the past one year?’	>1
Nutrition		
Body mass index	Weight (kg)/height^2^ (meter)	<18.5 kg/m^2^
Unintentional weight loss	Proportion of weight lost as compared to the pre-illness baseline	>10%
Nutritional status	MNA [28]	<24 (24–30: Normal nutrition; 17–23.5: At risk of malnutrition; <17: Malnourished)
Comorbidities		
Presence of comorbidities	Hypertension, diabetes, chronic airway disease, coronary artery disease, any other comorbidity	Specific comorbidity is documented
Validated comorbidity tools	Charlson comorbidity index [29]	>2
Validated comorbidity tools	CIRS-G [30]	>4 Any category score: 3–4 Severity index (total score/number of scales with score >0) >2
Cognition		
Literate patients	MMSE [31]	<24 (24–30: no cognitive impairment; 18–23: mild cognitive impairment; 0–17: severe cognitive impairment)
Illiterate patients	Hindi mental status examination [32]	
Mood		
Depression	GDS-short form [33]	>4 (5–8: mild depression; 9–11: moderate depression; 12–15: severe depression)
Anxiety	GAD-7 [34]	>10 (0–4: minimal anxiety; 5–9: mild anxiety; 10–14: moderate anxiety; >15: severe anxiety)
Medications		
Polypharmacy	Number of regular medications	>5 medications [35]
PIM	American geriatrics society beers criteria [36]	Any PIM
Social support		
Validated tool	Older Americans resources and services medical social support [37]	>1 (in questions 2–4)
Living situation	Resident of Mumbai/staying with relative, or in a rented room or hotel/guest house of ashram/homeless/staying on the footpath	Homeless or staying on the footpath
Number of caregivers	‘How many persons are available to care for you all the time, or most of the time, or at the times that you need a caregiver?’	0
Screening tools		
	Geriatric 8 (G8) [38]	<12
	Vulnerable elders survey-13 (VES-13) [39]	>3
	Rockwood clinical frailty scale [40]	>5
Chemotherapy toxicity risk prediction score	CARG chemo-toxicity calculator [41]	Low risk (30%)*: 0–5 Intermediate risk (52%)*: 6–9 High risk (83%)*: >10 [42, 43]
Non-cancer life expectancy	ePrognosis (Lee and Schonberg indices) [44]	Estimate of the 5-year and 10-year mortality (non-cancer related) and the life expectancy in years
QoL	The European Organisation for Research and Treatment of Cancer QLQ-C30, v3.0 [45]	Higher score indicates a better QoL
	EORTC QLQ-ELD14 [46]	Higher scores on ‘Maintaining purpose’ scale and ‘Family support’ item indicates a high level of functioning; higher scores for ‘Mobility’, ‘Worries about others’, ‘Future worries’, ‘Burden of illness,’ and ‘Joint stiffness’ indicate a poor QoL
Caregiver QoL	(*Fronto* temporal lobe disorders) Caregiver burden scale	>20 (0–20: little or no burden; 21–40: mild to moderate burden; 41–60: moderate to severe burden; 61–88: severe burden)
General assessments		
Vision		
Distant vision	Snellen chart	6/18 or worse
Near vision	Landolt C chart	N/18 or worse
Hearing		
Questionnaire	Hearing handicap index [47]	>10 (0–8: no handicap; 10–24: mild to moderate handicap; 26–40: severe handicap)
Voice	Voice handicap index [48]	>11
Oral health	Geriatric oral health assessment index [49]	Total score: 12–60; higher scores indicate better perception of oral-dental health
Cancer-related symptoms	Edmonton symptom assessment scale [50]	Any score >4
Financial burden		
	Comprehensive score for financial toxicity-functional assessment of chronic illness therapy (COST-FACIT) [51]	<12 (≥26: no impact on QoL (grade 0); 14–25: mild impact (grade 1); 1–13: moderate impact (grade 2); 0: high impact (grade 3))
	Consumer financial protection bureau financial well-being scale [52]	Range: 0–100 points; Lower scores represent worse financial well-being
Patients’ and caregivers’ viewpoint regarding disclosure	‘Does the patient/caregiver want the treating team to disclose and discuss the diagnosis and prognosis?’	Based on the patients’ and caregivers’ viewpoints, the results of the GA, the curability/treatability of the cancer and the potential toxicity of the therapy, a discussion occurs
Patients’ expectations from treatment	If curative: Complete cure/symptom control/improvement in present status/improved QoL or prolongation of life If palliative: Improved QoL/prolongation of life/immediate symptom relief	
Blood tests		
	eGFR	Based on the kidney function, a decision will be taken
	NLR	NLR <3.4 = Poor prognosis
Vaccination status	Uptake of pneumococcal, influenza, and COVID-19 vaccines	If not received, patients are advised these vaccines

* Risk of grade 3 or higher toxicity

**Table 2.** Some of the research studies (completed, ongoing and planned) to bridge the knowledge gap of geriatric oncology in India.

Research/Study category	Study	Sample size, and time frame of the study	Key question	Status of the study	Key results	Impact
Epidemiologic research	Epidemiology and demographic profile of older Indian persons with cancer*	266,640 patients from 16 institutes across India; Jan 2019 to Dec 2020	- What proportion of Indian patients with cancer are aged 60 years and over? - What are the common types of cancers in older patients with cancer?	Study is complete; Manuscript in process of submission	- 36.4% of adult Indian patients with cancer were aged 60 years and over. - Common malignancies included gastrointestinal malignancies (23.9%), head-and-neck (18.8%), breast (10.5%), genitourinary (10.5%), lung (10.22%), haematological (8.8%).	- This study will help plan various policies, focus areas and other initiatives
Tool (GA tools) validation	CARG score validation in patients receiving curative intent therapy [64]	*N* = 270; 2021	Does the CARG score reliably predict grade 3 to 5 chemotherapy toxicities in geriatric patients treated with curative intent chemotherapy?	Completed, published	- 52% of patients had at least one grade 3–5 toxicity. - Risk of toxicities based on the CARG risk score: Low risk-42% - Intermediate risk-51% - High risk-79%; *p* < 0.001	CARG chemotherapy risk prediction score is valid and reliable in older Indian patients with cancer receiving curative intent therapy.
	G8 and VES-13 as screening tools and predictors of survival [65]	*N* = 308; Jun 2018 to Nov 2020	-What is the diagnostic accuracy of the G8/VES-13 screening tools to detect an abnormal GA? - What is the optimal cut-off value of G8 for older Indian patients with cancer? - Is an abnormal G8 associated with shorter survival?	Completed, published	**G8 (cutoff ≤14):** Sensitivity: 84.4% Specificity 17.6% - **VES-13 (cut-off >3):** Sensitivity: 34.9% Specificity: 82.4% - G8 ≤14 was not associated with abnormal GA (*p* = 0.736), or worse survival outcomes. - G8 <12 was associated with an abnormal GA (*p* < 0.001), and predicted for worse survival.	- G8 cut-off <12 is appropriate in older Indian patients with cancer. - G8 <12 is predictive of non-oncological vulnerabilities and shorter survival. - Lowering G8 cutoff to 12 translated to a 35% reduction in the number of patients undergoing a GA. This may help in optimal resource utilisation.
	TUG as a predictor of mortality [57]	*N* = 851; Jun 2018 to Jan 2022	- Is the TUG score associated with overall survival in older Indian patients with cancer? - What is the optimal cut-off for TUG in older Indian patients with cancer?	Completed, published	- TUG predicted mortality (HR, 1.058; 95% CI, 1.039–1.078). - Median overall survival of patients with TUG <12 seconds was 13.9 months (95% CI, 11.2 to 16.5), compared to 8.5 months (95% CI, 6.6 to 10.3) in those with a TUG ≥12 seconds (*p* = 0.002). -TUG cut-off score >10 seconds: AUC-ROC sensitivity: 62.3%, specificity 80.6%.	TUG can be a reliable tool in a busy outpatient setting to identify vulnerable patients who require a detailed GA. -TUG ≥10 seconds was a good predictor of impaired mobility.
	CARG-IND: Multicentric, double-blinded, prospective observational cohort study to validate the CARG score as a predictor of ≥grade 3 chemotherapy toxicities in older Indian patients with cancer receiving chemotherapy [66]*	N = 588; Started Dec 2022, ongoing	- Does the CARG score accurately predict the incidence of grade 3–5 chemotherapy-induced toxicities in older patients with cancer receiving systemic therapy?	Active recruitment ongoing	Pending	- Results of this study will help guide optimal systemic therapy decision-making in older patients with cancer receiving chemotherapy, including palliative intent therapy.
	Assessment of PIMs in older Indian patients with cancer [67]	N = 352; 2018 to 2021	- Which is the most reliable screening tool for assessment of PIM use in older adults with cancer?	Completed; manuscript submitted	- 5 PIM tools were compared and assessed: Beers-2015, STOPP and START-2014, PRISCUS-2010, FORTA-2018, and EU (7)-PIM list-2015 - EU (7)-PIM list had the least bias of 0.7% and the narrowest limits of agreement of 0.43 (−0.21 to 0.22). - PIM use was significantly higher in patients with diabetes (p = 0.013) and in patients taking >7 medications (p < 0.001)	- EU (7)-PIM list-2015 should be the tool of choice for assessing PIMs in older Indian patients with cancer. - However, high degrees of discordance were noted between the various tools used to assess PIMs, emphasising the need for local tools to be developed.
	GA as a predictor of survival in older Indian patients with cancer [68]	*N* = 897; Jun 2018 to Jan 2022	- Do the results of the GA correlate with survival in older Indian patients with cancer? - Which individual domains of the GA are predictive of survival?	Completed, manuscript submitted	- 85% of patients were frail (>1 impaired domain in GA) - GA was predictive of OS; - Median OS in fit patients was 24.3 (95% CI, 18.2-not reached) months, versus 11.2 (10.1–12.8) months in the frail patients; HR, 0.54; 95% CI, 0.41–0.72, *p* < 0.001 - Function, nutrition and cognition were individually predictive of survival on the multivariate analysis.	- GA is prognostic for survival in older Indian patients with cancer. - GA is prognostic even in patients thought to be the fittest, i.e., PS 0 and 1. - GA must be done in all older Indian patients planned for cancer-directed therapy.
Implementation research	SCOPE-C; version 1: The Screening of the older PErsons with cancer, Version 1-development and validation of a new screening tool for older adults with cancer in resource-limited settings [54, 55]	N = 419; May 2013 to Feb 2016	- Can a targeted assessment be performed in older patients with cancer in resource-limited settings like India where there is paucity of evidence in this field? - Can the SCOPE-C screening tool be converted into a prognostic one by providing score cut offs predictive of survival at 24 weeks?	Completed and published	- SCOPE-C consisting of 13 questions with sub-parts (35 items in total) was developed and validated on a sample of 100 subjects. - Cronbach's alpha: 0.93 and the intra-class correlation co-efficient was 0.94. - Time to administer the tool: 25 minutes - Male sex, functional decline, cognitive impairment, malnutrition, and treatment modality were independently associated with survival. - Individual scores on SCOPE-C V.1 correlated with 24-week survival - Cutoff score of 64 had a 72.2% sensitivity and 77.3% specificity for better prognosis.	- SCOPE-C is valid and reliable. - Preliminary assessment with SCOPE-C may help streamline care in resource-limited settings.
Descriptive/observational research	Impact of the GA on systemic therapy plan [60]	*N* = 617; Jun 2018 to Sept 2021	- In older patients with cancer, do the results of a GA lead to a change in the cancer-directed systemic therapy plan?	Completed, and published	- Systemic therapy plan was changed in 239 (38.7%) patients following the GA. - Most common change was treatment deintensification in 198 (32.1%) patients.	- GA is very important to optimally plan therapy in older Indian patients with cancer. - GA must be done before starting therapy in all older Indian patients with cancer.
	PIM use and polypharmacy [59]	*N* = 285; Jun 2018 to Oct 2020	- What is the the prevalence of polypharmacy, and PIM use in older Indian patients with cancer?	Completed, and published	- Polypharmacy (>4 medicines) noted in 55% patients - Excessive polypharmacy (>9 medicines) in 13%. - Polypharmacy noted in 70% patients with lung cancer, versus 45% for other malignancies, *p* < 0.001. - Unindicated medications such as vitamins and calcium taken by 20% patients - Alternative medicines (ayurvedic/homeopathic/naturopathic) taken by 23% - 80% of the patients were on PIMs, commonly proton-pump inhibitors (33%) and tramadol (30%).	- Polypharmacy and PIM use are common problems in older Indian patients with cancer. - We are in the process of implementing a widespread safe medication prescribing program based on this study.
	ECOG PS as representative of deficits in older Indian patients with cancer [6]	N = 594; May 2018 to Jan 2021	- Does the ECOG PS correlate with the individual GA components and with the burden of deficits as estimated in the GA?	Completed, and published	- ECOG PS ≥1 was predictive of abnormalities in >2 geriatric domains; AUC = 0.69 (95% CI, 0.64–0.74), sensitivity = 95.4%, specificity = 18.4% - With each 1 unit increase in ECOG PS, odds of having ≥2 geriatric abnormalities increased by 4.69 (95% CI, 2.53–8.68). - Median number of impaired geriatric domains based on PS: PS 0 = 1 (IQR, 1–2); PS 1 = 2 (IQR, 1–3); PS 2 = 3 (IQR, 2–4); PS 3 = 4 (IQR, 3–4). - ECOG PS correlated moderately well with deficits in cognition (AUC = 0.66 (95% CI, 0.61–0.72)), function and falls (AUC = 0.73 (95% CI, 0.69–0.77)), and psychological domains (AUC = 0.65 (95% CI, 0.60–0.70)) and poorly correlated with nutritional status (AUC = 0.63 (95% CI, 0.58–0.68)) and comorbidities (AUC = 0.55 (95% CI, 0.49–0.61)).	- Older patients with cancer with an ECOG PS ≥1 are very likely to harbor non-oncological vulnerabilities, and should therefore undergo a GA.
	Inflammatory markers and correlation with survival [69]	N = 787; Jun 2018 to Nov 2021	- Can the NLR, lymphocyte monocyte ratio (LMR) and platelet lymphocyte ratio (PLR) be used to predict overall survival in older Indian patients with cancer?	Completed, manuscript in process	- High NLR (OS: 9.1 versus 16.9 months; HR: 1.5, 95% CI; 1.3–1.8) and high PLR (OS: 9.3 versus 17.1 months; HR: 1.6, 95% CI; 1.3–2.0) were predictors of poor OS, - High LMR (OS: 14.2 versus 10.3 months; HR = 0.8, 95% CI; 0.7–0.9) was a predictor or better OS, even after adjusting for age, sex and primary tumour.	- With simple blood tests like the complete blood count, inlflammatory markers like NLR, PLR and LMR can be used to help prognosticate older Indian patients with cancer. - In combination with other clinicopathologic features, these may be used to help generate an individualised care plan for older Indian patients with cancer.
	Utilisation of technology among older Indian patients with cancer [70]	N = 309; Apr 2021 to Oct 2021	- How many older patients with cancer and their caregivers use mobile phones, Internet and social media applications? - Does the use of technology have any association with various factors?	Completed, and published	- 81% patients had mobile phones. - 25% patients used the Internet - 21% patients used some form of social media (10%: Whatsapp only; 11%: Whatsapp and Facebook) - 99% caregivers used mobile phones - 75% caregivers used E-mail and social media applications. - Women and those with no education, poor vision, and impaired cognition were less likely to own a mobile phone. - People with no education and impaired cognition were less likely to use Internet and social media.	- Although the use of Internet and social media applications was low among our patients, the high utilisation of technology by the caregivers supports the vision of technology use to provide healthcare at the doorstep. - Development of an online GA screening tool would likely be possible given the almost universal use of technology by caregivers, which could help bridge some of the limitations, including time taken for evaluation, requirement of additional staff and bridge the gap between health care provider and older Indian patients with cancer.
	Vaccination uptake in older Indian patients with cancer [71]	- What is the uptake of pneumococcal, influenza, and coronavirus- 2019 (COVID-19) vaccines among older patients with cancer? - Are there any factors associated with vaccination uptake?	N = 1,762; Feb 2020 to Jan 2022	Completed, and published	- 0.68% patients had received pneumococcal vaccine - 0.7% had received influenza vaccine. - 83.3% had received at least one dose of the COVID-19 vaccine. - Factors associated with COVID-19 vaccine uptake: education, marital status, geographic zone and primary tumour site.	- Fewer than one in 100 older Indian persons with cancer receive routine immunisation with influenza and pneumococcal vaccines. - The uptake of COVID-19 vaccination in older Indian persons with cancer is over 80% - Similar strategies as those used to increase COVID-19 vaccination should be employed to increase the uptake of routine vaccinations.
	Real-world experience on the use of immune checkpoint inhibitors (ICIs) for solid tumours in older adults with cancer [72]	What are the treatment-related outcomes and toxicities of ICIs in older Indian patients with cancer?	N = 150; Aug 2014 to Feb 2021	Completed, and published	- Common indications for ICI: NSCLC (52.7%) and HNSCC (17.3%). -Nivolumab was commonest ICI used in 119 (79.4%) patients. - ICIs were used in the palliative setting in 144 (96%) patients. - Median number of ICI cycles: 5 (IQR, 3.0–9.5). - ORR to ICIs: 30%, clinical benefit rate: 52%. -Median PFS: 4.23 months (95% CI, 1.38–7.08) months - Median OS: 8.6 months (95% CI, 4.9–12.2) - Baseline PS was the most significant prognostic factor for PFS and OS in the multivariate analysis.	- ICIs are well tolerated in older Indian patients with cancer, with no new safety concerns. - ICI’s appear to be efficacious in older Indian patients with cancer. - Additional prospective studies to assess the role of ICIs, and the contribution of immunosenescence are needed.
	Financial toxicity among older Indian patients with cancer [73]	- What is the level of financial toxicity in older Indian persons with cancer? - Is there a correlation betweem financial toxicity and QoL? - What factors are associated with financial toxicity?	*N* = 498; Jun 2022 to Jan 2023	Completed, manuscript in process	- Source of funding for cancer-therapy: family members (53.9%), insurance (14.7%), self pay (12%). - 12.4% had to borrow money, 4.3% had taken a loan, 2.6% sold their assets for the purposes of treatment. - Moderate-severe financial burden on COST-FACIT present in 33.1%, whereas 66.9% experienced no or mild burden. - High financial toxicity was associated with lower socioeconomic status (OR: 4.69, 95% CI 2.47–8.91, *p* < 0.001), poor financial well-being (OR: 9.35, 95% CI 5.52–16.46, *p* < 0.001) and poor score on Financial QoL (OR: 5.16, 95% CI 3.39–7.86, *p* < 0.001)	- There is high prevalence of financial burden in older Indian patients with cancer - Financial toxicity is associated with poor socioeconomic status, poor financial well-being and poor financial QoL. - Recognition of financial toxicity is important to optimally plan and choose the most appropriate therapy options.
	ReproGeri study: Representation of older Indian patients with cancer in interventional clinical trials at the Tata Memorial Hospital [74]	- What is the representation of older Indian patients with cancer in interventional clinical trials at the Tata Memorial Hospital, Mumbai? - How many studies have eligibility criteria that limit the enrollment of older Indian persons with cancer?	N = 21,443; 2005 to 2022	Completed, manuscript in process	- 149 interventional clinical trials at the TMC - Median age of all enrolled patients: 51 years (IQR, 43–59). - Of all patients enrolled in interventional clincal trials: 23.5% were >60 years, and 4.8% were >70 years. - In the same timeframe, 30.6% of adult patients registered in the hospital were aged >60 years. - In 2.84% studies, an upper age limit was part of eligibility criteria - In 26.2% studies, ECOG PS 2 was an exclusion criterion - In 63.8% trials, uncontrolled comorbidities were exclusion criteria.	- There is an underrepresentation of older Indian adults with cancer in interventional clinical trials. - Increasing the representation of older adults with cancer in clinical research is critical to establishing the optimal cancer-directed therapy in this group of vulnerable individuals.
Therapeutic/interventional research	Optimising chemotherapy in the first line for older patients with advanced non-small cell lung cancer (Phase III randomised non-inferiority study) [75]	- Can a lower dose of standard cytotoxic chemotherapy in older persons with advanced non-small cell lung cancer result in lower toxicity, while maintaining survival?	*N* = 308; Active enrolment ongoing	Enrolling	Pending	- If a lower dose of chemotherapy is found to be non-inferior as compared to full dose chemotherapy, this will help improve the risk-benefit ratio, and will enhance the patients’ therapeutic experience.
	CARGO: A randomised phase III clinical trial evaluating the non-inferiority of reduced dose chemotherapeutic regimens based on CARG risk scores compared to standard doses in older patients with advanced esophageal, esophagogastric, gastric, and biliary tract cancers [76]	- Does a chemotherapy dose reduction based on the CARG chemotherapy risk score lead to similar OS as full dose standard chemotherapy in older patients with advanced biliary tract, esophageal, esophago-gastric, and gastric cancers?	*N* = 410	Enrolling	Pending	- If the study proves that dose reduction based on the CARG risk prediction score leads to non-inferior survival, this may result in the uniform use of reduced chemotherapy doses in older patients with cancer, thus improving the therapeutic index.
	GOCoG: Geriatric oncology multidomain intervention study to prevent cognitive impairment among older Indian patients with cancer receiving chemotherapy: a randomised controlled trial (GOCoG)	- Can a 3-month multidomain intervention (cognitive training + exercise) decrease the chemotherapy-related cognitive decline in older Indian patients with cancer receiving chemotherapy, compared to usual care?	N = 364	Submitted to the ethics committee of the Tata Memorial Hospital, Mumbai; awaiting approval	Pending	If the intervention succeeds in improving the chemotherapy-related cognitive decline in older Indian patients with cancer, this will have a significant impact on the patients in terms of: - QoL - functional status - independence - decision-making capacity - treatment compliance
	Effects of prehabilitation on outcomes in older Indian patients with cancer: a randomised controlled study	-Will a short-term multimodal prehabilitation program with exercise, respiratory training, nutrition consultation, anxiety reduction anemia correction optimisation of comorbidities and medicines, and smoking cessation reduce postoperative complications and length of hospital stay in older Indian patients with cancer?	Protocol being planned	Protocol being submitted to the ethics committee of Tata Memorial Hospital, Mumbai	Pending	- If the study is positive, it will support the implementation of prehabilitation in all older patients planned for radical cancer-directed surgery. - A reduction in post-operative complications and a decrease in the length of hospital stay will be important and will help improve the therapeutic ratio of surgery.
	Phase III open label superiority randomised trial comparing concurrent chemoradiotherpay to radiation therapy alone in older patients with locally advanced HNSCC	- Does the addition of concurrent chemotherapy to radical intent radiation improve oncologic outcomes in older patients with locally advanced HNSCC	Protocol being planned	Protocol being submitted to the ethics committee of Tata Memorial Hospital, Mumbai	Pending	The results of this study will help us to decide the optimal regimen for patients with locally advanced HNSC planned for non-surgical curative therapy options.
	Optimal assessment of renal function in older Indian patients with cancer [77]	- Which equation for calculating the estimated GFR can most accurately estimate the renal function in older Indian patients with cancer?	N = 276; Jun 2021 to Nov 2022	Completed, manuscript in process	- GFR was estimated with six formulae: Cockcroft-Gault (CG); six variable MDRD; six variable MDRD; CKD-EPI creatinine; CKD-EPI cystatin; CKD-EPI creatinine-cystatin; BIS 1; and BIS 2 equations. - GFR was also measured by ^99m^Tc-DTPA plasma technique (Gates’ method). - Serum cystatin estimated by sandwich ELISA - BIS 2 equation had the least bias (0.854) and narrowest 95% LoA (−31.23 to 32.94) had the highest agreement with ‘gold standard’. - BIS 2 performed consistently well across ages (60–70, 71–80, 81–90) and BMI categories (≤18.5, 18.6–24.9, ≥25)	- Equations derived using cystatin C in general, and BIS 2 in particular, have highest predictive accuracy for GFR estimation in older patients with cancer. - Accurate assessment of renal function is essential particularly in patients receiving renally cleared or nephrotoxic chemotherapy.
Patient-and cergiver-centric research	Caregiver burden experienced by the caregivers of our older patients with cancer [78]	- What is the burden experienced by caregivers of our older patients with cancer?	*N* = 127; Jun 2020 to Dec 2020	Completed, published	- Caregiver burden was assessed using the Zarit Burden Interview - Median caregiver burden score: 12 (IQR, 6–20) - Caregiver burden was little/none in 76.4%, mild-moderate in 19.7%, moderate-severe in 3.1%, and severe in 0.8% - Psychological issues in the patient and the caregivers’ educational level significantly impacted the caregiver burden scores.	- Caregiver burden was low in our study - By identifying the cohort of patients most likely to have higher caregiver burden, targeted interventions could be planned to help these caregivers, and lessen the burden.
	Patients’ expectations from therapy, and disclosure of diagnosis/prognosis [79]	- Do older Indian patients with cancer wish to know the details of their diagnosis and prognosis? - What are patients' goals from cancer-directed therapy?	*N* = 319; 2018 to 2021	Completed, manuscript in progress	- 83% patients wished to know about their diagnosis and prognosis. - In 19% cases, the caregivers did not want the disease-related details to be disclosed to the patients. - 85% patients in the curative setting expected a complete cure - 63% patients in the palliative setting valued an improved QoL.	- Recognising patients’ and caregivers’ perspectives is of paramount importance in shared decision making. - It is important to understand that two-thirds of the patients being treated with palliative intent therapy valued a maintained QoL over a prolonged life.
	QoL in older Indian patients with cancer [80]	- How are the different QoL domains affected in treatment-naïve older patients with cancer?	N = 360; July 2015 to Jun 2017	Completed, published	- Fatigue was reported in 68.9% patients, loss of appetite in 66.4% and pain in 50%. - Poor functioning (score <50) was seen in 66%) patients in the global QoL domain (overall health and QoL in the preceding one week), in 68.9% in the role functioning domain, in 55.5% in the physical functioning domain, and in 74.3% in the emotional functioning domain. -Financial constraints were reported by 66.4% patients.	- Understanding the QoL issues in older patients with cancer, particularly with regards to which individual domains are likely to be affected will help in planning early interventions, targeted at improving the QoL of these patients.

* Collaborative multicentric study

Acronyms: CARG = Cancer and Ageing Research Group; TUG = Timed Up and Go test; PIM = Potentially inappropriate medicines; GA = Geriatric assessment; QoL = Quality of life; ECOG PS = Eastern Cooperative Oncology Group; PS = performance status; AUC = Area under the curve; CI = Confidence interval; OS = Overall survival; HR = Hazard ratio; COST-FACIT = COmprehensive Score for financial Toxicity‐Functional Assessment of Chronic Illness Therapy; OR = Odds ratio; GFR = Glomerular filtration rate; IQR = Interquartile range; NSCLC = Non-small-cell lung cancer; HNSCC = Head and neck squamous cell carcinoma; IQR = Interquartile range; CI = Confidence interval

**Table 3.** Action points and implementation strategies to take geriatric oncology in India forward in the next decade.

Focus areas	Goal	Possible implementation methods
Increasing the uptake of the GA throughout India	Every older Indian patient with cancer should undergo a GA with targeted interventions prior to planning cancer-directed therapy.	• Continuing education regarding the importance of GA • Using technology (application-based GAs) • Developing a short India-specific GA • Use of screening tools like G8 and VES
Building a cadre of professionals trained in geriatric oncology	There should be specialists who are trained in the assessment and care of older patients with cancer at every institution and clinic that manages older persons with cancer.	• Robust training programs to provide education in the principles and practice of geriatric oncology • Ongoing and frequent hands-on workshops and webinars
Inter-instutional collaboration	To establish an inter-institutional collaborative network of healthcare professionals for knowledge-sharing, collaborative research and other multicentric activities.	• To establish and register the Geriatric Oncology Society of India. • Hold monthly meetings online • Organise an annual meeting/conference to decide the purpose, planned work and manifesto of the society. • Overarching goal will be to enhance the quality of service, education, and geriatric oncology research across multiple institutions in India
Multicentric research studies	To generate evidence that answers the unique issues of older Indian patients with cancer.	• Establish a group of like-minded researchers within the collaborative oncology network • Build a clinical trials wing within the group, and establish the necessary manpower and infrastructure to conduct high-quality research studies. • Brainstorm regarding the top research priorities, and decide the most important focus areas. • Apply to various funding agencies for the top projects.
India-specific geriatric oncology guidelines	To establish a set of minimum and optimal guidelines to streamline the assessment and care of older Indian patients with cancer	• In order for these guidelines to be relevant and applicable to older Indian patients with cancer, we will need to: • Modify the existing global guidelines, and/or • Generate new culturally appropriate and country specific tools for assessment, and interventions. • These guidelines will have to be updated on a regular basis, perhaps every 2 to 3 years.

Acronyms: GA = geriatric assessment, G8 = Geriatric 8; VES = Vulnerable elders survey
